# Indications, contraindications, and step-by-step methodology for performing carotid sinus massage in patients presenting with syncope: A scientific statement of an Ad Hoc Syncope Consortium endorsed by: the European Autonomic Society (EFAS), Gruppo Italiano Multidisciplinare Syncope (GIMSI) and European Geriatric Medicine Society (EuGMS)

**DOI:** 10.1007/s10286-026-01218-z

**Published:** 2026-05-27

**Authors:** Frederik J. de Lange, Michele Brignole, David G. Benditt, Melani Dani, Jean Claude Deharo, Alessandra Fanciulli, Artur Fedorowski, Antonella Groppelli, Mohamed H. Hamdan, Viktor Hamrefors, Jelle S. Y. de Jong, Rose Anne Kenny, Piotr Kulakowski, Angel Moya, Roberto Maggi, Carlos A. Morillo, Brian Olshansky, Satish R. Raj, Ciara Rice, Giulia Rivasi, Ineke van Rossum, Vincenzo Russo, Robert Sheldon, Win-Kuan Shen, Richard Sutton, Roland Thijs, Patricia Taraborrelli, Marco Tomaino, Andrea Ungar, Steven van Zanten

**Affiliations:** 1 Department of Clinical and Experimental Cardiology, Amsterdam Cardiovascular Sciences, Heart Centre, University of Amsterdam, Meibergdreef 9, 1105AZ Amsterdam, The Netherlands; 2 Department of Cardiology Milan, IRCCS Istituto Auxologico Italiano, Milan, Italy; 3 Cardiovascular Division, Department of Medicine, University of Minnesota Medical School, Minneapolis, MN USA; 4 Department of Bioengineering Cutrale Perioperative and Ageing Group, Imperial College London, London, W12 0BZ UK; 5 Assistance Publique, Hôpitaux de Marseille, Service de Cardiologie, Centre Hospitalier Universitaire La Timone, Marseille, France; 6 Aix Marseille Université, C2VN, 13005 Marseille, France; 7 Department of Neurology, Medical University of Innsbruck, 6020 Innsbruck, Austria; 8 Department of Cardiology, Karolinska University Hospital, Department of Medicine, Karolinska Institute, Stockholm, Sweden; 9 Department of Clinical Sciences, Lund University, Malmö, Sweden; 10 Division of Cardiovascular Medicine, Department of Medicine, School of Medicine and Public Health, University of Wisconsin, Madison, WI USA; 11 Department of Cardiology, Skåne University Hospital, Malmö, Sweden; 12 Trinity College and St James Hospital, Dublin, Ireland; 13 Department of Cardiology, Centre of Postgraduate Medical Education, Grochowski Hospital, 04-073 Warsaw, Poland; 14 Department of Cardiology, Hospital Universitari Dexeus, 08028 Barcelona, Spain; 15 Department of Cardiology, Ospedali del Tigullio, Lavagna, Italy; 16 Libin Cardiovascular Institute, Cumming School of Medicine, University of Calgary, Calgary, AB Canada; 17 Division of Cardiology, Department of Medicine, University of Iowa Carver College of Medicine, Iowa City, IA USA; 18 Department of Experimental and Clinical Medicine, University of Florence, Florence, Italy; 19 Division of Geriatric and Cardiogeriatric Medicine, Careggi University Hospital, Florence, Italy; 20 Department of Neurology, Leiden University Medical Centre, Leiden, the Netherlands; 21 Department of Translational Medical Sciences, Cardiology and Syncope Unit, University of Campania ‘Luigi Vanvitelli’, Monaldi Hospital, Piazzale E. Ruggeri, 80126 Naples, Italy; 22 Department of Cardiovascular Diseases, Mayo Clinic Arizona, Scottsdale, USA; 23 Department of Cardiology, National Heart and Lung Institute, Imperial College London, Hammersmith Hospital Campus, London, UK; 24 Stichting Epilepsie Instellingen Nederland (SEIN), Heemstede, The Netherlands; 25 Imperial Syncope Unit, Imperial College Healthcare NHS Trust, London, W12 0HS UK; 26 Division of Cardiology, Ospedale Generale Regionale, Bolzano, Italy; 27 Department of Cardiology, Reinier de Graaf Gasthuis, Delft, The Netherlands

**Keywords:** Syncope, Carotid sinus massage, Diagnostic, Clinical practice

## Abstract

Carotid sinus massage (CSM) is essential in evaluating recurrent unexplained reflex syncope, as it uniquely identifies cardioinhibitory forms that other tests may miss. CSM is largely underused in clinical practice outside dedicated syncope facilities and, when performed, its execution varies, as does the interpretation of results. Underuse affects the diagnostic yield of CSM in the syncope evaluation, thereby denying patients mechanism-based personalized treatment. There are several barriers to the proper use of CSM in the work-up of patients with unexplained syncope.

To address these important limitations, an international panel of experts in the field of syncope provide herein a consensus document with the aim of offering practical guidance for the indications, contraindications, and methodology for performing carotid sinus massage in general and dedicated syncope facilities and in the emergency department.

## Introduction

Carotid sinus massage (CSM) has been used for over a century, primarily to interrupt supraventricular tachycardia [1, 2]. Since the late 1970s, it has been utilized as a diagnostic tool for cases of unexplained syncope [2] and to assist in guiding cardiac pacing treatment [3—6].

Current guidelines of the European Society of Cardiology (ESC) for the management of syncope clearly delineate the indications for CSM, interpretation of findings, and role in treatment selection [7, 8]. These guidelines recommend CSM in patients > 40 years of age with syncope of unknown origin and compatible with a reflex mechanism (class I, B) [7] and recommend cardiac pacing therapy in patients with cardioinhibitory carotid sinus syndrome who suffer from recurrent and unpredictable syncope and in whom there is reproduction of symptoms with CSM (class I, A) [7].

CSM is essential in evaluating recurrent unexplained reflex syncope, as it uniquely identifies cardioinhibitory forms that other tests may miss [9]. CSM is largely underused in clinical practice outside dedicated syncope facilities [9] and, when performed, its execution varies as does the interpretation of CSM results. Underuse of CSM affects the diagnostic yield of the syncope evaluation and thereby denies patients mechanism-based personalized treatment [9].

The role of CSM has been better defined only during the last decade. The test is better performed with the patient on a tilt table with noninvasive continuous beat-to-beat blood pressure monitoring, ideally in the same session as tilt testing, to detect vasodepressor or hypotensive responses [9]. However, CSM can also be performed in general outpatient clinics or emergency departments without noninvasive continuous beat-to-beat BP monitoring or a tilt table, but this limits assessment to the cardioinhibitory aspect only.

## Barriers to the implementation of carotid sinus massage in clinical practice

Currently, there are several barriers to the proper use of CSM in the work-up of patients with unexplained syncope:

### Operator dependency

The execution of CSM is still largely operator-dependent, with inter-operator variability in indications and interpretation of results [10]. Massage pressure can differ significantly between practitioners, affecting results. Although duration is easy to standardize, pressure and finger placement are harder to measure objectively. Incorrect positioning or insufficient pressure may cause false negatives and misleading conclusions.

### False perception of the risks (liability issues)

The fear of causing irreversible damage to the patient is still a reason that many physicians refrain from performing CSM. This is combined with the risk of subsequent liability issues. This belief stems from literature reports prior to the year 2000, which showed an already low and clinically acceptable complication rate of 3.6/1000 patients (11—14). More recently, three large series indicated that the complication rate has greatly decreased by more than tenfold to 3.0/10,000 (15—17). Considering that all the events were completely reversible, these observations make CSM a very safe procedure (Table 1) [10]. The decrease in complication rate in the most recent literature may be explained by better risk assessment in current practice for older patients with unexplained syncope [18] and by improved preventive and treatment strategies for stroke [19] together with more careful selection of patients with unexplained syncope in whom CSM is performed [7].

**Table 1:** Complication rate for carotid sinus massage before and after the year 2000 (adapted from de Lange et al.) [10]

Older studies (performed before the year 2000)						
	Inclusion period	*N* (patients)	Age, mean ± SD	Male, %	TIA/stroke (%)	Specification of complication
Puggioni et al. Am J Cardiol 2002 [11]	1996–2000	1719	63 ± 16	56%	3 (0.17%)	TIAs < 24 h in 3
Davies et al. Am J Cardiol 1998 [12]	1993–1998	4000	74 ± 14	na	11 (0.27%) (*)	TIAs < 24 h in 9, persistent hemiparesis in 1, visual field loss in 1
Munro et al. JAGS 1994 [13]	na	1600	na	na	7 (0.44%) (**)	TIAs < 24 h in 4, stroke from 1 to 7 days in 3
Richardson et al. Age Ageing 2000 [14]	na	1000	69 ± 10	31%	9 (0.9%) (***)	TIAs < 24 h in 8, mild weakness of right hand > 24 h
Total		8319			30 (0.36%)	
					3.6/1000	

Most recent studies (performed after the year 2000):						
	Inclusion period	*N* (patients)	Age, mean ± SD or median (IQR)	Male, %	TIA/stroke (%)	Specification of complication
Torabi et al. Europace 2023 [15]	2008–2021	1634	64 ± 13	39%	0	0
Brignole et al. Europace 2020 [16]	2003–2019	3293	73 (64–80)	48%	2 (0.06%)	TIAs < 24 h in 2
Ungar et al. Age Ageing 2016 [17]	na	1401	72 ± 16	41%	0	0
Total		6328			2/6328 (0.03%)	0
					3 /10,000	

TIAs: transient ischemic attacks; SD: standard deviation; na: not available; IQR: interquartile range. (*) Davies: 2 patients had had previous TIA/stroke > 6 months beforehand. Carotid Doppler normal in 9, stenosis 70% in 2. (**) Munro: 1 patient had 70% carotid artery stenosis, (***) Richardson: Doppler ultrasound normal in 8, carotid stenosis 50% in 1

### Lack of reimbursement

CSM itself is not independently reimbursed in many healthcare systems (e.g., public and private). Limited reimbursement can discourage clinicians from performing CSM, especially in productivity-driven or risk-averse environments. As a result, they may opt for alternative diagnostics like prolonged cardiac monitoring, which are reimbursable but often more costly and less immediately informative. The recently published 2STEPS protocol [9] provides an efficient and straightforward diagnostic approach for evaluating syncope in patients over 40 years of age, incorporating CSM into the head-up tilt table test (HUTT), a procedure reimbursed by many healthcare systems. However, in areas where HUTT reimbursement is not available, there is a further challenge to performing CSM due to the requirement for access to a suitably equipped autonomic testing laboratory staffed by trained professionals [20].

### Unawareness of clinical benefit of CSM

Although CSM is a straightforward procedure, it remains underutilized in general outpatient clinics and emergency departments, in part because of the mistaken belief that advanced monitoring methods, such as noninvasive continuous beat-to-beat blood pressure measurement, are necessary. Although less desirable, CSM can be effectively performed in an outpatient clinic and emergency department using only ECG monitor recording [21].

Failing to use CSM in outpatient and emergency settings leads to missed diagnoses of carotid sinus syndrome (CSS), a treatable cause of syncope and falls in older adults. Without CSM, many cases of reflex syncope remain undetected, increasing the risk of recurrent syncope, falls, and injuries including fractures and head trauma, causing repeat visits, increasing emergency care, hospital admissions, and decreased quality of life. Identifying CSS early through CSM can prevent these outcomes [22]. Pacemaker therapy, which is effective in cardioinhibitory CSS [23], may thereby be delayed or not considered at all. Patients may undergo costly or invasive tests that are often ineffective or delay diagnosis, such as neurological imaging, unnecessary ECG-Holter monitoring, or inappropriate placement of an implantable loop recorder (ILR) that lead to increased healthcare utilization and attendant costs.

#### Low visibility of CSM in diagnostic flowcharts and guidelines

The low visibility of CSM in diagnostic flowcharts and guidelines contributes directly to its underuse in clinical practice. For example, in the American College of Cardiology (ACC)/American Heart Association (AHA) guidelines [24], CSM is not mentioned under the section “additional evaluation and diagnosis.” These guidelines mention tilt table testing as a Class IIa indication when a reflex mechanism is suspected, but CSM is not mentioned at all. As a result, many US physicians are not aware of the role of CSM in the evaluation of syncope when a reflex mechanism is suspected. Raising the profile of CSM in clinical pathways, especially in outpatient clinics and SUs, is essential for improving the diagnosis and management of reflex syncope.

#### Absence of clear rules for performing CSM for allied professionals

In several countries, allied health care professionals such as nurse practitioners, physician assistants, trained nurses, or cardiovascular technicians have an increasingly important role in the management of the dedicated syncope facilities and syncope unit (SU) [25, 26] by performing the initial evaluation, HUTT [25], initiating monitoring, and even undertaking invasive tests and treatment as ILR and pacemakers, respectively. On the other hand, CSM may not be performed by allied health care professionals, probably because of the fear of inherent risk/complication induced by the maneuver. This is clearly a contradiction, likely due to ancient practice rather than hard data. The primary obstacle to allied professionals using CSM is the absence of comprehensive rules, training modules, and credentialing. This scientific statement aims to outline relevant scientific data and suggest potential regulatory guidelines to support the use of CSM by allied professionals (see the section “Integration into clinical practice”).

## Scope of this scientific statement

There is a clear need to establish indications, contraindications, and step-by-step methodology for performing CSM in patients presenting with syncope.

To that end, we established the Ad Hoc Carotid Sinus Syncope Consortium, a multidisciplinary team of experts in syncope who shared concerns about current CSM practices. The consortium is endorsed by the European Autonomic Society (EFAS), Gruppo Italiano Multidisciplinare Syncope (GIMSI).

This document reflects the opinion of the group and is aimed at promoting awareness, discussion, and future research in the field, but is not a formal evidence-based clinical guideline. The two chairs took the responsibility of identifying the panel of international experts based on their recognized expertise and authority in the topic and wrote a first draft of the text that was submitted to the panel for revision. Based on comments received, the chairs replied point-by-point to the comments of the panel and wrote a second draft which was again submitted for a second round of revision. In the case of discordance of opinion regarding important issues, the chairs moderated formalized back-and-forth negotiations according to the evidence-based consensus methodology called “Delphi consensus,” and a voting system was used to reach consensus.

In this document, we provide practical guidance on when to use CSM, how to perform and interpret CSM, and strategies to improve its application across syncope facilities, general clinics, and emergency departments. This statement also supports educational programs to train healthcare professionals in accurately performing CSM. Table 2 outlines the recommended protocol.

**Table 2:** Protocol for performing the carotid sinus massage

Purpose: To evaluate CSM as a potential diagnostic test in unexplained syncope in adults > 40 years
Personnel qualification
Any physician who is experienced with syncope management
Trained allied professional e.g., nurse practitioners, physician assistants, trained nurses, syncope technicians in accordance with national guidelines and hospital-specific protocols
It is recommended that both physician and allied professional have passed a quality self-assessment evaluation (see proper section) and completed competency validation
II. Equipment and environment
Motorized tilt table with fast tilt-down time [12 s or less] [41]
ECG monitor with continuous recording and ECG tracing which should be retrievable for review
Noninvasive continuous beat-to-beat BP monitor with continuous recording which should be retrievable for review and maintained in the protocol record
Emergency resuscitation equipment (including external pacer/defibrillator pads)
Stopwatch/timer (optional)
Documentation/charting system (electronic medical report, EMR) (optional)
III. Indications
Unexplained recurrent syncope or unexplained falls in patients > 40 years of age with syncope of unknown origin but compatible with a reflex mechanism
Prior negative work-up for orthostatic hypotension, and exclusion of cardiac cause of syncope or non-syncopal transient loss of consciousness
IV. Contraindications
History of TIA, stroke within the last 3 months
Medical history is not compatible with reflex syncope
*Note:* In general, avoid performing CSM if there is no potential benefit of performing the massage

V. CSM procedure steps (according to the Six-Step Method, see video [10]) Supine phase CSM (both in general outpatient clinic or emergency department and dedicated syncope facility/SU)
Position patient supine on bed (general outpatient clinic or emergency department) or on the tilt table (in dedicated syncope facilities /SU)
Observe 1–2 min baseline HR and BP
Turn head slightly away from massage side
Massage carotid sinus: just below angle of mandible, anterior to sternocleidomastoid on level of superior ridge of cricoid cartilage (see video Six-Step method [10])
Right side first for 10 s
Wait until HR and SBP stabilize
Repeat on left side, if appropriate (see termination criteria)
Continue real-time monitoring and observe diagnostic criteria
Tilt phase CSM (only in dedicated syncope facility/SU) or autonomic/clinical physiology laboratory
Tilt patient to 60°–70° head-up and allow 3–5 min for stabilization
Repeat CSM on each side as above, if appropriate (see termination criteria)
VI. Diagnostic criteria
Reproduction of spontaneous symptoms in presence of
Cardioinhibitory response: asystole ≥ 3 s
Vasodepressor response: SBP fall ≥ 50 mmHg in absence of asystole ≥ 3 s
*Note.* To identify the presence of a vasodepressor component typically linked with the cardioinhibitory form (termed the “mixed” form), CSM should be repeated following the administration of 0.02 mg/kg atropine [7, 8]

VII. Termination criteria
The test can be terminated when the above diagnostic criteria are met
It is appropriate to continue massage on the other side or body position until completion of the protocol, even when the first massage is diagnostic, in order to assess the presence of contrasting forms (i.e., vasodepressive form in case of previous cardioinhibitory form or vice versa)
VIII. Final clinical report (chart or EMR). See example in Appendix II
Indication
Baseline vitals, monitoring modality
CSM side, sequence, and position (supine/upright)
Hemodynamic responses and symptoms
Diagnostic interpretation
Complications and interventions (if any)
IX. Quality control and supervision
Checklist audit for procedural accuracy
Immediate physician review of positive findings

*BP* blood pressure, *CSM* carotis sinus massage, *HR* heart rate, *SBP* systolic blood pressure, *SU* syncope unit

## Criteria for positive/negative response of CSM

According to the 2018 European Society of Cardiology (ESC) syncope guidelines [7, 8], CSS is confirmed in patients with unexplained recurrent syncope in whom CSM causes reproduction of spontaneous symptoms in the presence of asystole (> 3 s) and/or hypotension (> 50 mmHg drop of systolic BP), and patients have clinical features compatible with a reflex mechanism of syncope.

Reproduction of syncope during CSM implies that the patient can recognize that the symptoms induced during the massage are similar to those of the spontaneous episodes. In the case that the patient does not remember the circumstances before fainting (absence of prodromes or retrograde amnesia), induction of syncope is considered a positive response.

Cardioinhibitory CSS is diagnosed when symptoms are present during an asystole lasting > 3 s. Vasodepressor CSS is diagnosed when symptoms occur in the absence of asystole > 3 s but with a fall in blood pressure > 50 mmHg. Mixed CSS is when both criteria are met on CSM (> 3 s asystole and BP fall > 50 mmHg). In the case of a mixed response, determining the relative contribution of cardioinhibition and vasodepression requires the repetition of the procedure after administration of 0.02 mg/kg i.v. atropine to prevent bradycardia but exposes the BP fall due to vasodepression alone [27].

When CSM is asymptomatic or unable to reproduce symptoms but elicits an asystole ≥ 3 s (cardioinhibitory response), or systolic blood pressure drop ≥ 50 mmHg (vasodepressor response), or both (mixed response), the term “carotid sinus hypersensitivity” (CSH) is used [7, 28]. CSH is common in older adults without a history of syncope. Thus, CSH due to CSM has low specificity [29, 30], therefore is not useful for diagnosing syncope and on its own does not warrant treatment intervention. However, in a small group of patients with an established clinical diagnosis of reflex syncope, the presence of CSH with an asystolic pause > 3 s was able to predict an asystolic spontaneous syncope documented by ILR in 89% of patients [31]. A 76% positive predictive value of asystolic CSH has recently been confirmed in a larger population [32], thus suggesting a potential role of CSH in identifying a bradycardic phenotype and guiding the subsequent management.

### Treatment of carotid sinus syndrome

When the patient is severely symptomatic, treatment is justified. Cardioinhibitory CSS implies the need for permanent pacing [23]. Cardioneuroablation may become a promising alternative, but its current application is premature, and further studies are necessary [33]. Vasodepressive CSS requires management to prevent blood pressure drop. Treatment requires interventions in order to prevent hypotensive episodes and increase the baseline blood pressure in patients with ambient low blood pressure or for those intensively treated with medications specifically used to lower the blood pressure to treat hypertension [33].

## Clinical indications and contraindications: risk–benefit assessment

### Indications for CSM

The primary indications for CSM are as follows:

- To establish the cause of unexplained syncope or unexplained falls (i.e., falls with no evident accidental cause such as slipping or tripping) of suspected reflex origin in patients over age 40 [7], when the initial syncope evaluation fails to reveal a definitive diagnosis
- To establish the mechanism (bradycardic or hypotensive) of recurrent reflex syncope when a mechanism-specific treatment is recommended

CSM may also be safely performed in patients under age 40 years, even if in these patients CSM is rarely abnormal in these patients [7, 8], unless the neck has been subject to prior surgery and/or radiation. The rationale for this age cutoff is based on the known reduced baroreceptor sensitivity and increased CSH prevalence with advancing age [11].

The benefit of CSM lies in its potential to establish the cause for and mechanism of reflex syncope, thus contributing to initiation of mechanism-based specific therapy [10].

**Fig. 1:**
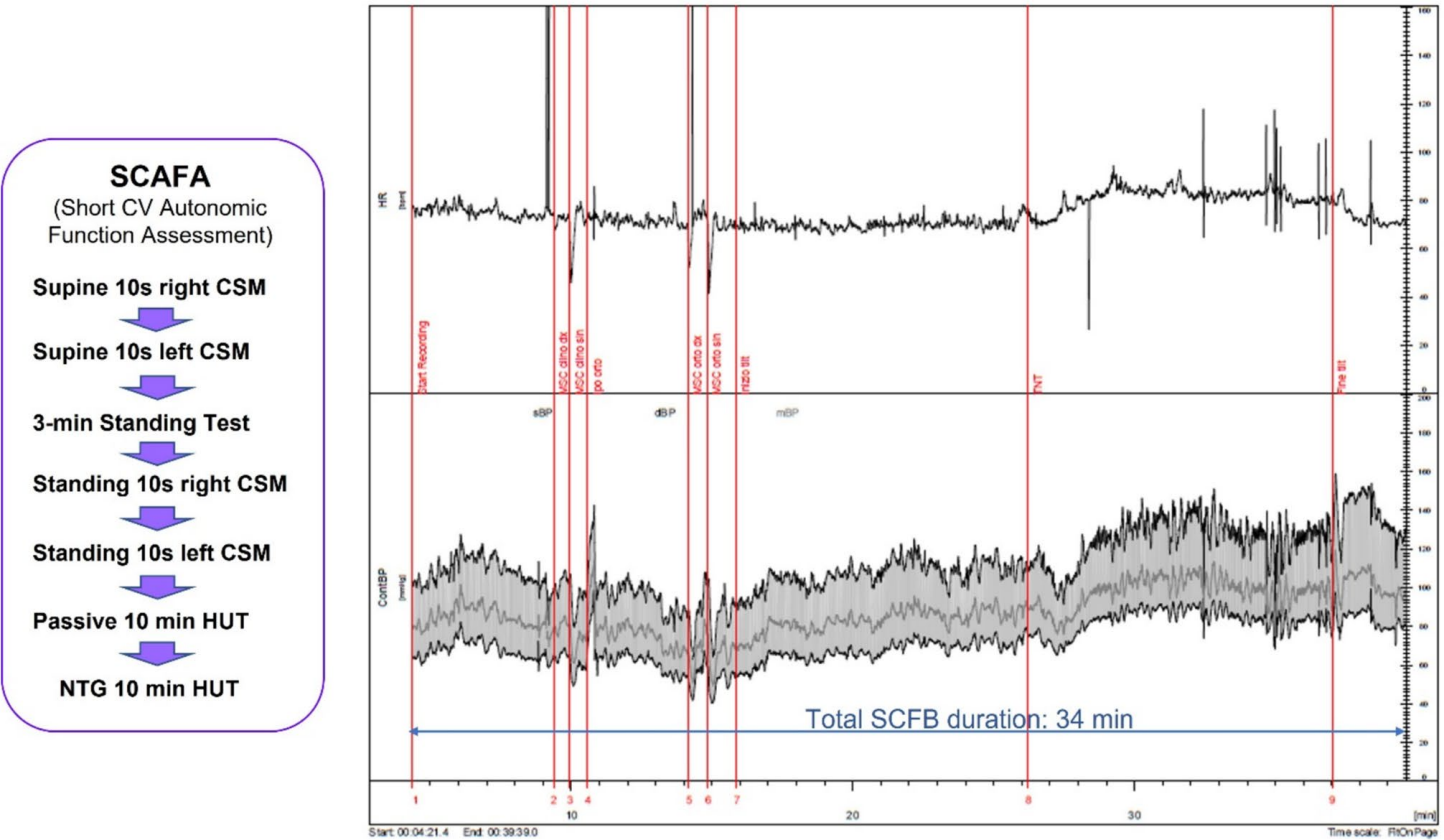
Exemplary case of positive CSM and positive HUTT (**A**) and negative CSM and negative HUTT (**B**) during SCAFA test performed on a tilt table during continuous beat-to-beat monitoring of heart rate (ECG) and systolic, mean, and diastolic blood pressure (photoplethysmographic method). The top panel shows the heart rate trend. The bottom panel shows blood pressure trend. The sequence of tests (red vertical lines) was as follows: (1) Supine, rest; (2) Supine, right carotid sinus massage (CSM); (3) Supine, left CSM; (4) Passive standing test; (5) Standing right CSM; (6) Standing, left CSM; (7) Head-up tilt test (HUT), passive phase; (8) HUT, nitroglycerine phase; (9) tilt-down. The supine right and left CSM caused hypotension and bradycardia without reproduction of spontaneous symptoms. The standing right CSM caused syncope recognized by the patient. The nitroglycerine HUT phase ended with a mixed form of vasovagal syncope with hypotension and bradycardia without asystole > 3 s. Reprinted with permission from Groppelli et al. [9]

**Fig. 2:**
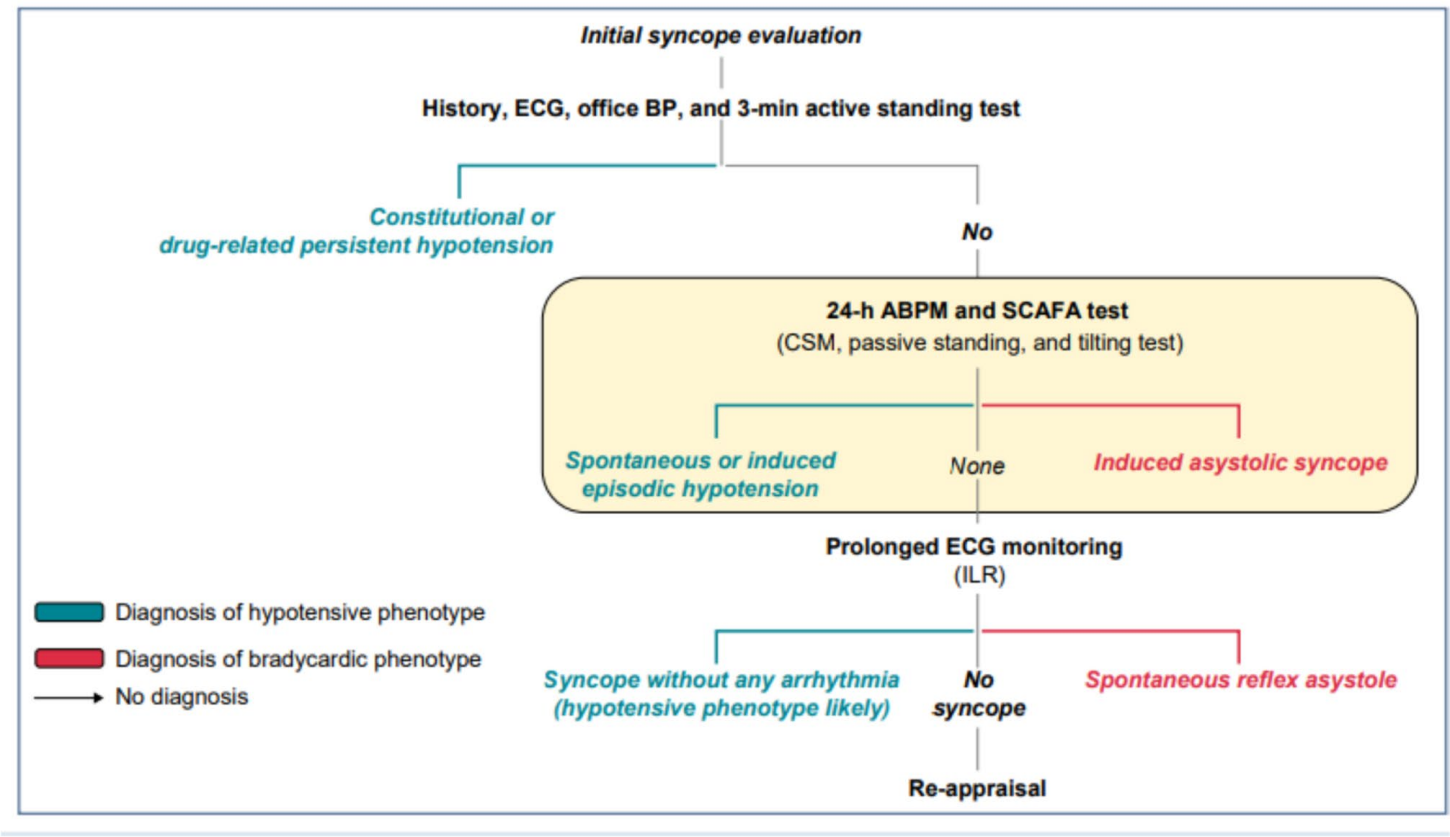
Diagnostic work-up of autonomic syncope by mechanism. The initial evaluation consists of history taking, standard 12-lead ECG, and automated blood pressure measurement supine and during 3 min of active standing. When the results of the initial evaluation are unremarkable, no additional testing is recommended, and treatment should start. When a diagnosis is not clear and/or in the case of recurrences after the initial evaluation and subsequent treatment, the second phase consists of 24 h ambulatory blood pressure monitoring and short cardiovascular autonomic assessment (SCAFA) that includes CSM and tilt testing performed sequentially during noninvasive continuous ECG and beat-to-beat BP monitoring. When the history indicates orthostatic symptoms right after standing up (initial orthostatic hypotension [iOH]) or upon prolonged standing (delayed orthostatic hypotension [dOH]), adaptations to the SCAFA test battery should be considered, including first a (prolonged) standing test preferably under beat-to-beat monitoring (iOH/dOH) or prolonged HUTT without CSM or Nitro after 10 min (dOH). Finally, an implantable loop recorder is usually needed if uncertainty persists on the mechanism of syncope. Reprinted with permission from Groppelli et al. [9]

Dual pathology (i.e., concomitant delayed orthostatic hypotension and reflex syncope) is not uncommon in older patients [15], where syncope events suggest a more reflex mechanism which cannot be explained solely by orthostatic hypotension. Therefore, CSM is particularly effective when combined with HUTT as described [34—37] because comprehensive syncope evaluation is completed in a single session including supine and upright CSM. Noninvasive continuous beat-to-beat BP with ECG monitoring is required to perform CSM combined with HUTT; see exemplary case Fig. 1A, B and Fig. 2 for a practical flow guide for personalized mechanism-based therapy of autonomic syncope.

**Fig. 3:**
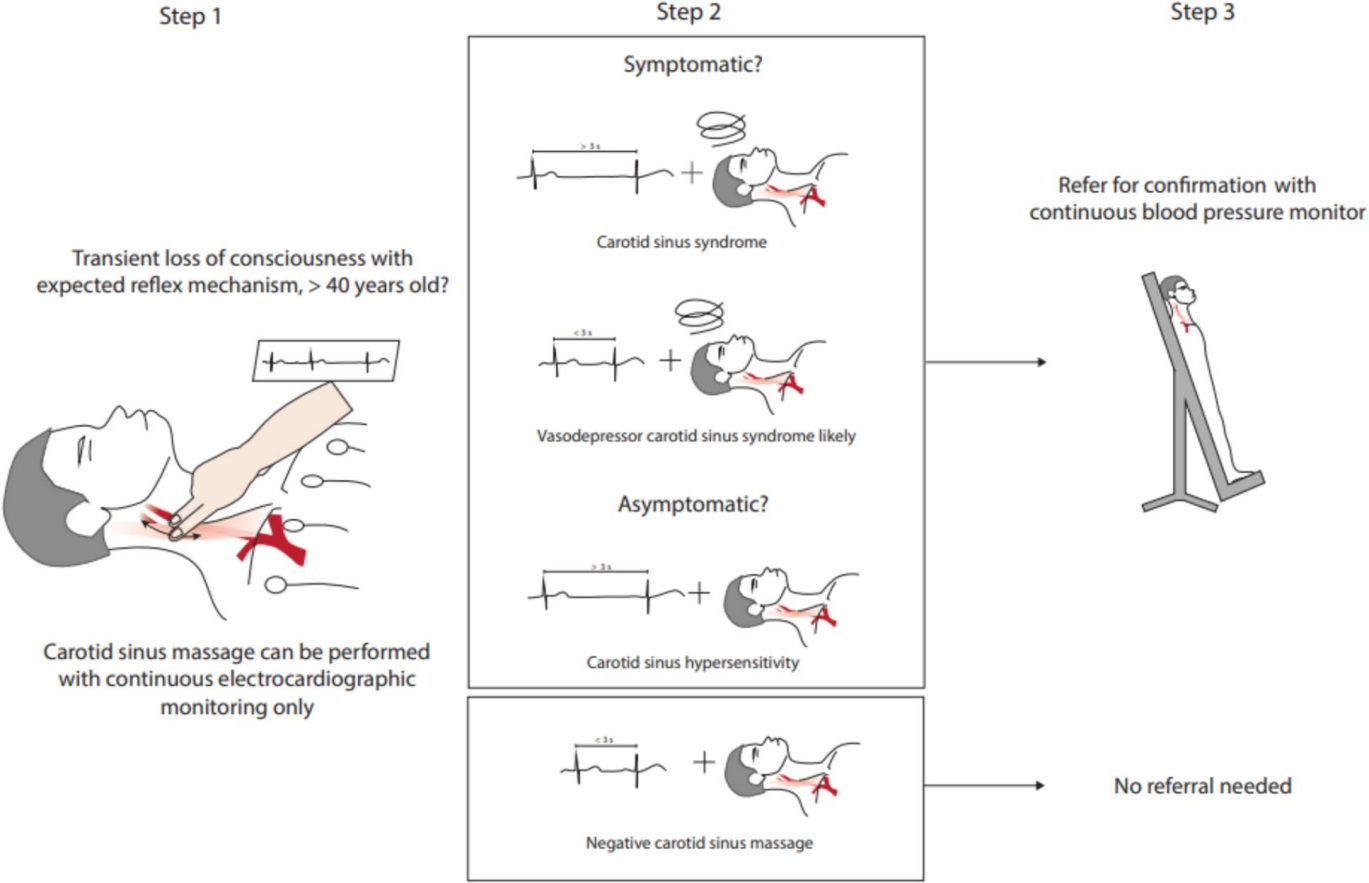
Explanatory figure for Table 3 of CSM outcomes when performed without noninvasive continuous beat-to-beat blood pressure monitoring (continuous ECG tracing required)

However, in a general outpatient clinic or emergency department evaluation, CSM is acceptable even without continuous beat-to-beat BP monitoring and without HUTT as part of the initial evaluation (Table 3 and Fig. 3).

**Table 3:** Interpretation of CSM without noninvasive continuous beat-to-beat blood pressure monitoring (continuous ECG tracing required)

Finding	Interpretation
Pause ≥ 3 s + reproduction of symptoms	Suggests cardioinhibitory CSS*
Pause ≥ 3 s, no reproduction of symptoms	Indicates CSH*
No pause, reproduction of symptoms	Vasodepressor CSS likely*
No pause, no symptoms	Negative CSM

* In the case of a positive or inconclusive response, referral to a dedicated syncope facility for a complete protocol with continuous beat-to-beat BP monitoring is recommended

### Contraindications to CSM

CSM is generally safe, as recent pooled analysis showed a low and clinically acceptable absolute risk (Table 1) [10]. The risk of TIA/stroke secondary to CSM is not different from the risk caused by performing carotid echo Doppler [38]. The risk may be minimized if the method of performing CSM is standardized according to the best practice recommended by current guidelines. These recommend a complete initial syncope evaluation in every patient with syncope, including careful history taking and physical examination [7]. The risk–benefit ratio should be evaluated for each patient. Usually, the benefit of performing CSM outweighs the risk, particularly when a diagnosis of CSS can lead to effective targeted treatments, such as pacemaker implantation for cardioinhibitory CSS.

CSM should not be performed in the case of the following:

- History of TIA or stroke within the last 3 months
- Medical history is not compatible with reflex syncope

Over 50 years of global CSM use shows no evidence that carotid artery stenosis increases the risk of complications during CSM, as most events occurred in patients without stenosis (Table 1). Thus, history of carotid stenosis or bruits does not represent a contraindication for CSM but warrants careful TIA/stroke risk assessment. Therefore, routine pre-CSM Doppler screening is not recommended.

## Integration into clinical practice

Integrating CSM into the routine clinical practice of a dedicated syncope facility or SU requires a structured, interdisciplinary, and guideline-driven approach that ensures safety, diagnostic utility, and operational efficiency [9, 37].

CSM has been shown to be particularly effective when it is performed as part of the 2STEP assessment including 24-h ambulatory blood pressure monitoring (ABPM) and short cardiovascular autonomic function assessment (SCAFA) [9]. SCAFA consists of CSM on both sides in supine and standing position, passive standing test, and a “fast” HUTT performed sequentially during one session on a tilt table (Fig. 1) [9]. This two-step protocol is an easy-to-perform and time-saving diagnostic work-up which allows the identification of the hemodynamic mechanism of loss of consciousness in 90% of patients with autonomic syncope [9]. If such evaluation is inconclusive, the next phase is prolonged ECG monitoring by implantable loop recorder (Fig. 2).

### The role of the allied professionals

Allied health care professionals (AHCPs) including nurse practitioners, physician assistants, nurses, or cardiovascular technicians may perform CSM if properly trained and qualified, although any attending physician who can help should remain available on-site in the case of a rare emergency. The attending medical doctor should retain ultimate responsibility for ongoing care [25, 26]. This requires clear institutional policies, resolution of liability concerns, structured training modules (may include entrusted professional activities), and credentialing in accordance with national and hospital policies.

### The role of CSM performed outside the syncope facility/SU without noninvasive continuous beat-to-beat blood pressure monitoring

CSM is a useful, low-tech diagnostic tool in outpatient and emergency settings when only an ECG trace is available (from standard ECG machine or telemetry). A continuous, reviewable ECG must be recorded during the procedure and recovery. Typically, CSM is performed with the patient supine. If symptoms are reproduced—with or without asystolic pause—or CSM revealed an inconclusive response, referral to a syncope facility is recommended to confirm the diagnosis and complete the assessment of the vasodepressor component, which requires continuous beat-to-beat blood pressure monitoring (Table 3 and Fig. 3).

This panel advises against promptly implanting a pacemaker after CSS-induced syncope until the full diagnostic assessment is complete, as syncope recurs in roughly 25% of patients within 3 years due to an undetected vasodepressor cause [21, 39].

In some outpatient clinics, intermittent manual cuff blood pressure measurement can be paired with ECG tracing, and CSM may be performed both supine and standing. Two professionals—typically a physician and nurse—can work together, one conducting the massage and the other quickly measuring systolic blood pressure during and after CSM. This approach improves diagnostics by revealing vasodepressor and cardioinhibitory reflexes that might not appear with supine-only massage.

**Ethical considerations**

From a legal perspective, CSM involves a low but absent procedural risk, especially in older adults or those with vascular disease [10]. We therefore recommend the following:

- Counsel the patient before CSM, explaining possible temporary symptoms and rare complications like stroke (< 0.03% incidence; see Table 1). An example consent form, if local practice warrants such procedure, is provided in Appendix I.
- Involve a credentialed physician or a certified allied professional supervised by a physician performs the procedure.
- Document the procedure including indication, patient screening, procedure details, hemodynamic response, symptoms, adverse events, and patient information provided (Appendix II).
- When non-physician allied personnel carry out this procedure, it is necessary to have an established protocol, a credentialing system, and remote oversight by a physician.

Medicolegal protection is ensured through adherence to established guidelines, careful patient selection, and comprehensive documentation. Institutions are recommended to incorporate CSM protocols within dedicated syncope facilities or SU and implement ongoing training and quality assurance evaluations.

CSM should be documented and audited within syncope management quality programs, recording patient demographics, procedure details, hemodynamic responses, symptoms, complications, and outcomes. Institutions can use this information to assess diagnostic yield and improve care pathways.

## Quality self-assessment

The method of performing CSM is still largely operator-dependent, with consistent inter-operator variability in results of the CSM [9, 10]. Operators can assess their skill by performing right supine CSM on 20 consecutive syncope patients. Competency is demonstrated by achieving a median increase in max R-R interval of at least 200 ms, or an increase of 200 ms in at least 10 cases. This threshold is based on data from 2249 syncope patients [40].

## Conclusions

Carotid sinus massage is a useful and safe diagnostic tool that should be more widely considered in the evaluation of unexplained syncope, especially in older adults. Its effectiveness depends on careful risk assessment, proper timing within evaluation, adherence to protocols, trained personnel, and informed consent.

## Abbreviations

ABPM: Ambulatory blood pressure monitoring
AST: Active standing test
BP: Blood pressure
CSM: Carotid sinus massage
ECG: Electrocardiogram
EHRA: European Heart Rhythm Association
ESC: European Society of Cardiology
HUTT: Head-up tilt test
SU(s): Syncope unit(s)
T-LOC: Transient loss of consciousness
ILR: Implantable loop recorder
